# Relationship between lifestyle and metabolic factors and carotid atherosclerosis: A survey of 47,063 fatty and non-fatty liver patients in China

**DOI:** 10.3389/fcvm.2022.935185

**Published:** 2022-08-12

**Authors:** Chun Zhang, Jiangang Wang, Siqing Ding, Gang Gan, Lijun Li, Ying Li, Zhiheng Chen, Yinglong Duan, Jianfei Xie, Andy S. K. Cheng

**Affiliations:** ^1^Health Management Center, The Third Xiangya Hospital, Central South University, Changsha, China; ^2^Xiangya Nursing School, Central South University, Changsha, China; ^3^The Third Xiangya Hospital, Central South University, Changsha, China; ^4^Department of Rehabilitation Sciences, The Hong Kong Polytechnic University, Hong Kong, Hong Kong SAR, China

**Keywords:** lifestyle, metabolic factors, carotid atherosclerosis, fatty liver, large sample

## Abstract

**Background and aims:**

Carotid atherosclerosis and stenosis are common lesions of the artery wall that form the basis of cardiovascular events. Compared with coronary atherosclerosis, few studies have explored the influencing factors of carotid atherosclerosis. The aim of this study was to explore the influencing factors of carotid atherosclerosis and carotid stenosis without and with fatty liver disease (FLD).

**Methods:**

A total of 47,063 adults were recruited for this cross-sectional study. The color Doppler ultrasound, including metabolic factors and lifestyle surveys, was used to determine whether the participants had FLD and carotid artery disease. Multiple logistic regression was used to investigate the influencing factors of lifestyle and metabolism of carotid atherosclerosis and stenosis in the participants with and without FLD.

**Results:**

In participants without FLD, current alcohol consumption (OR: 0.749, 95% CI: 0.588) and hip circumference (OR: 0.970, 95% CI: 0.961, 0.979) were the main protective factors for carotid atherosclerosis. Systolic blood pressure (OR: 1.022, 95% CI: 1.019, 1.025) and diastolic blood pressure (OR: 1.005, 95% CI: 1.001, 1.010), elevated fasting blood glucose (OR: 1.012, 95% CI: 1.005, 1.019), and non-sedentary behavior (OR: 1.084, 95% CI: 1.014, 1.160) were the main risk factors for carotid atherosclerosis. Hip circumference (OR: 0.932, 95% CI: 0.910, 0.954) and low-density lipoprotein (OR: 0.979, 95% CI: 0.964, 0.994) were protective factors for carotid stenosis. Smoking (OR: 3.525, 95% CI: 1.113, 11.169) and unqualified exercise (OR: 1.402, 95% CI: 1.083, 1.815) were risk factors for carotid stenosis. In participants with FLD, smoking (OR: 0.827, 95% CI: 0.703, 0.973) and hip circumference (OR: 0.967, 95% CI: 0.958, 0.977) were the main protective factors for carotid atherosclerosis. BMI 18.5–23.9 (OR: 1.163, 95% CI: 1.002, 1.351), non-sedentary behavior (OR: 1.086, 95% CI: 1.009, 1.168), and waist circumference (OR: 1.030, 95% CI: 1.022, 1.038) were the main risk factors for carotid atherosclerosis.

**Conclusion:**

Based on a large-sample check-up population in China, this study investigated the influencing factors of carotid atherosclerosis and carotid stenosis in fatty liver and non-fatty liver patients and explored the influencing factors of metabolism and lifestyle, which were mainly focused on exercise, sedentary behavior, smoking, alcohol consumption, hip circumference, and blood pressure.

## Introduction

Fatty liver disease (FLD) is a common chronic disease that is a pathological process of excessive accumulation of fat in liver cells caused by various factors, such as disease or drugs (1). With the development of the disease, fatty liver can progress from simple steatosis to steatohepatitis and can develop into cirrhosis in serious cases. FLD is becoming the most common liver disease in the world and is not only associated with significant morbidity but also leads to higher socioeconomic costs and impaired health-related quality of life (2). Previous studies have shown that fatty liver is often affected by lifestyle factors, such as diet and exercise (3). Hepatic steatosis has also been found to be associated with individual metabolic factors, including diabetes, hypertension, impaired fasting glucose, high-density lipoprotein cholesterol (HDL-C), and hypertriglyceridemia (4).

Cardiovascular disease (CVD) is one of the major public health problems threatening the health of Chinese people (5). Prevention of CVD risk factors and early diagnosis and treatment of high-risk groups can effectively reduce mortality. Carotid atherosclerosis and stenosis are common lesions of the artery wall that form the basis of CVD, which may lead to narrowing and occlusion of the arteries (6).

Studies have explored the relationship between FLD and coronary atherosclerosis and found that people with FLD have a higher probability of coronary atherosclerosis than those without FLD (4). Other studies have also found that the increased risk of FLD was associated with cardiovascular risk factors and persisted after adjustment for overall obesity or visceral adipose tissue, suggesting a bidirectional relationship between fatty liver and cardiovascular risk factors (7). Moreover, in a study of 265 patients with early liver disease, carotid intima–media thickness (cIMT) was higher in patients with fatty liver than in those without fatty liver (8), and fatty liver was associated with increased cIMT, artery calcification, and endothelial dysfunction (9).

However, compared with coronary atherosclerosis, few studies have explored the influencing factors of carotid atherosclerosis among fatty and non-fatty liver patients. In our study, we conducted a large-sample survey to explore the lifestyle and metabolic factors affecting carotid atherosclerosis and stenosis in both participants with and without FLD to provide evidence for targeted prevention of carotid artery disease and further reduce the risk of CVD.

## Materials and methods

### Study design and participants

**Figure 1:**
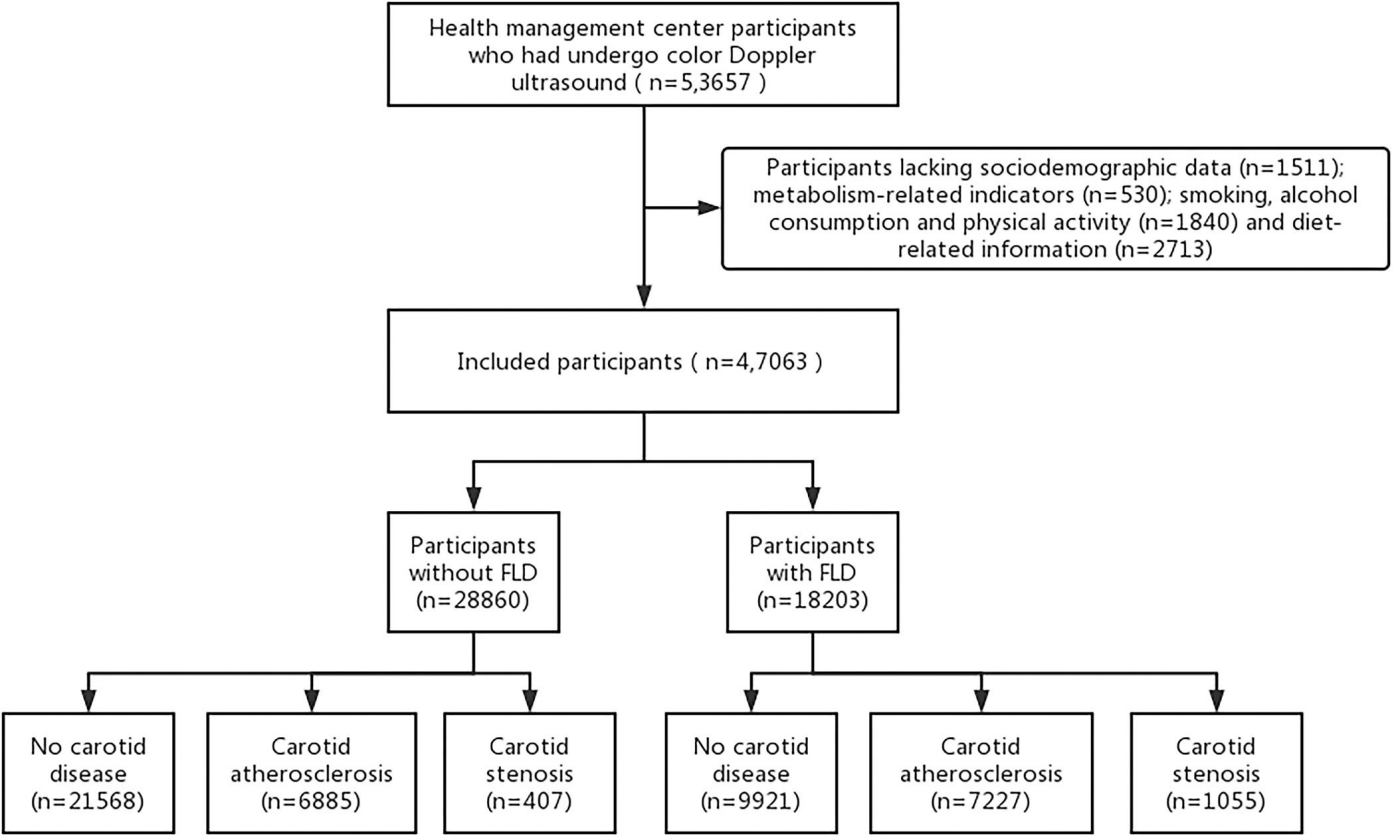
Flowchart of this study.

The study was a cross-sectional survey, and we recruited participants from two health management centers of general hospitals located in China. From 1 January 2017 to 31 December 2019, a total of 53,657 people aged ≥18 years underwent a color Doppler ultrasound of the liver and carotid artery. The following participants were excluded: participants who lacked sociodemographic data (n=1511) and metabolism-related indicators (*n* = 530) and participants lacking data on smoking, alcohol consumption, physical activity (*n* = 1,840), and diet-related information (*n* = 2,713). As a result, 47,063 participants were assessed in further data analysis. All the institutions involved in this study have given their approval. Informed consent was obtained from each participant in this study, and participation was voluntary without any reward (see Figure 1).

### Measures

#### Demographic and lifestyle characteristics

We collected the following data for each participant through questionnaires: sex, age, and body mass index (BMI), which was categorized as lean, normal weight, overweight, and obese for BMI < 18.5, 18.5–23.9, 24–27.9, and ≥28 kg/m^2^, respectively. Smoking was classified as a non-smoker, current smoker, and ex-smoker, while alcohol consumption was classified as none, yes, and abstinent from alcohol. Based on the American College of Sports Medicine's standards (10), physical activity standards were judged based on whether the amount of activity in the previous month was more than 12 times per month, including the intensity, duration, and frequency. The Dietary Diversity Scale (DDS) was used to evaluate dietary diversity, which was divided into nine categories according to the 2016 Edition of Chinese Residents' Balanced Diet Pagoda (11), including cereals, vegetables, fruits, livestock and poultry meat, fish and shrimp, eggs, milk, beans, and oil. According to the total number of types of food consumed by the subjects in a week, the consumption score is 1, and the non-consumption score is 0. The level of dietary diversification was divided into three grades: 1 to 3 was insufficient, 4 to 6 was moderate, and 7 to 9 was sufficient (12). Sedentary behavior was defined as waking, using the equivalent of 1.5 MET while sitting or lying down (13), and a metabolic equivalent was defined as the amount of energy expended while sitting at rest. According to the relevant literature (14), with 5 h/d as the cutoff, sedentary behavior was defined as ≥5 h/d, and non-sedentary behavior was defined as < 5 h/d.

#### Common metabolic risk factors

According to the standard of waist and hip circumference unique to Asia, waist circumference (WC) was measured by trained researchers at the midpoint between the base of the thoracic cage and the top of the iliac crest, and hip circumference was measured at the symphysis pubis and the most convex part of the posterior gluteus maximus while the subjects were standing. Blood pressure was measured by a trained nurse using a sphygmomanometer while subjects sat in a seated position with their arms supported at the heart level. Blood lipid examination results included fasting blood glucose, total cholesterol (TC), triglyceride (TG), HDL-C, and low-density lipoprotein cholesterol (LDL-C).

### Statistical analysis

All data in this study were collated and analyzed using SPSS 25.0. Demographic and lifestyle data were described by frequencies, and metabolic indicators were described by mean (M) ± standard deviation (SD). Multiple logistic regression was used to investigate the influencing factors of carotid atherosclerosis and stenosis in participants without and with fatty liver. Odds ratios (ORs) and 95% confidence intervals (CIs) were reported, with a test level of α = 0.05.

## Results

### Demographic characteristics and the prevalence of carotid atherosclerosis and stenosis

Of 47,063 participants, 28,860 (61.3%) had no FLD and 18,203 (38.7%) had FLD. The demographic characteristics and lifestyle characteristics of the participants are listed in Table 1, and metabolic factors are listed in Table 2. Among the people without FLD, 21,568 (74.7%) did not have carotid artery disease, 6,885 (23.9%) suffered from carotid atherosclerosis, and 407 (1.4%) suffered from carotid stenosis. There were 3,604 participants ≤ 30 years, accounting for 12.5%; 14,377 participants were 31–49 years old, accounting for 49.8%; 9,025 participants were 50–64 years old, accounting for 31.3%; and 1,854 participants were ≥ 65 years old, accounting for 6.4%. Among participants with FLD, there were 9,921 (54.5%) participants without carotid artery disease, 7,227 participants (39.7%) with carotid atherosclerosis, and 1,055 participants (5.8%) with carotid stenosis. There were 13,132 male (72.1%) and 5,071 female participants (27.9%). A total of 1,253 (6.9%) participants were ≤ 30 years, 8,843 participants (48.6%) were 31–49 years, 6,924 participants (38.0%) were 50–64 years, and 1,183 (6.5%) participants were ≥ 65 years.

**Table 1:** Demographic characteristics and lifestyle factors.

**Variable**		**Without FLD**		**With FLD**	
		**N**	**Percentage (%)**	**N**	**Percentage (%)**
Sex	Men	14,634	50.7%	13,132	72.1%
	Female	14,226	49.3%	5,071	27.9%
Age	≤ 30	3,604	12.5%	1,253	6.9%
	31–49	14,377	49.8%	8,843	48.6%
	50–64	9,025	31.3%	6,924	38.0%
	≥65	1,854	6.4%	1,183	6.5%
BMI	<18.5	1,134	3.9%	106	0.6%
	18.5–23.9	16,743	58.0%	4,274	23.5%
	24–27.9	9,123	31.6%	9,575	52.6%
	≥28	1,860	6.4%	4,248	23.3%
Smoking	Non-smoker	19,512	67.6%	9,592	52.7%
	Current	8,570	29.7%	7,771	42.7%
	Ex-smoker	778	2.7%	840	4.6%
Alcohol	Never	19,321	66.9%	9,185	50.5%
	Current	9,158	31.7%	8,665	47.6%
	Abstinent from alcohol	381	1.3%	353	1.9%
Physical activity	Unqualified	22,246	77.1%	14,222	78.1%
	Qualified	6614	22.9%	3,981	21.9%
Sedentary behavior	Yes	18,473	64.0%	11,107	61.0%
	None	10,387	36.0%	7,096	39.0%
Dietary diversity	Insufficient	2,187	7.6%	1,578	8.7%
	Moderate	6,344	22.0%	3,778	20.8%
	Sufficient	20,329	70.4%	12,847	70.6%
Carotid artery Disease	None	21,568	74.7%	9,921	54.5%
	Carotid atherosclerosis	6,885	23.9%	7,227	39.7%
	Carotid stenosis	407	1.4%	1055	5.8%

**Table 2:** Metabolic related factors.

**Variable**	**Without FLD**		**With FLD**	
	**M**	**SD**	**M**	**SD**
Waistline	79.26	9.322	87.896	8.9884
Hip circumference	95.77	553.457	96.466	10.0494
Systolic pressure	120.28	15.911	127.68	15.720
Diastolic pressure	73.07	10.884	79.42	11.177
FBG	5.57	3.977	6.24275	6.494533
Total cholesterol	6.18	6.892	6.0780	5.74361
TG	2.92	5.631	4.4457	7.81684
HDL-C	2.75	4.534	3.2967	5.11097
LDL-C	5.48	8.765	5.4248	8.68359

### Factors influencing carotid disease in participants without FLD

The results of multiple logistic regression analysis in participants without FLD are shown in Table 3.

**Table 3:** Multivariate logistic regression of influencing factors of carotid artery disease.

			**Without FLD**					**With FLD**				
			**B**	**SE**	**Wald**	**P**	**OR(95%CI)**	**B**	**SE**	**Wald**	**P**	**OR(95%CI)**
Carotid arteriosclerosis
	Intercept		−0.237	0.446	0.283	0.594		−1.009	0.487	4.283	0.038	
	Waistline		−0.001	0.003	0.125	0.724	0.999 (0.992, 1.006)	0.029	0.004	57.766	0.000	1.030 (1.022, 1.038)
	Hip circumference		−0.030	0.005	41.355	0.000	0.970 (0.961,0.979)	−0.033	0.005	43.090	0.000	0.967 (0.958,0.977)
	Systolic pressure		0.022	0.002	189.298	0.000	1.022 (1.019, 1.025)	0.013	0.002	52.115	0.000	1.013 (1.010, 1.017)
	Diastolic pressure		0.005	0.002	5.125	0.024	1.005 (1.001, 1.010)	0.005	0.003	3.487	0.062	1.005 (1.000, 1.010)
	FBG		0.012	0.004	10.361	0.001	1.012 (1.005, 1.019)	0.010	0.003	11.874	0.001	1.010 (1.004, 1.015)
	Total cholesterol		−0.002	0.002	0.506	0.477	0.998 (0.994, 1.003)	−0.005	0.003	3.152	0.076	0.995 (0.989, 1.001)
	TG		−0.003	0.003	1.272	0.259	0.997 (0.992, 1.002)	−0.002	0.002	1.154	0.283	0.998 (0.993, 1.002)
	HDL-C		−0.005	0.003	2.065	0.151	0.995 (0.988, 1.002)	0.002	0.003	0.249	0.618	1.002 (0.995, 1.008)
	LDL-C		0.002	0.002	1.194	0.274	1.002 (0.999, 1.005)	−0.003	0.002	1.782	0.182	0.997 (0.994, 1.001)
	Gender	Men	0.099	0.038	6.740	0.009	1.104 (1.024, 1.189)	−0.080	0.048	2.843	0.092	0.923 (0.840, 1.013)
		Female	0^b^					0^b^				
	Age	≤ 30	−2.579	0.096	720.595	0.000	0.076 (0.063,0.092)	−2.442	0.128	366.548	0.000	0.087 (0.068,0.112)
		31–49	−1.526	0.058	694.794	0.000	0.218 (0.194,0.244)	−1.359	0.072	359.836	0.000	0.257 (0.223,0.296)
		50–64	−0.061	0.055	1.250	0.264	0.940 (0.845, 1.047)	0.098	0.070	1.974	0.160	1.103 (0.962, 1.265)
		≥65	0^b^					0^b^				
	BMI	<18.5	−0.520	0.144	13.108	0.000	0.594 (0.448,0.788)	−0.040	0.316	0.016	0.898	0.960 (0.517, 1.785)
		18.5–23.9	−0.019	0.085	0.051	0.821	0.981 (0.830, 1.160)	0.151	0.076	3.945	0.047	1.163 (1.002, 1.351)
		24–27.9	−0.003	0.072	0.002	0.968	0.997 (0.865, 1.149)	0.107	0.052	4.156	0.041	1.112 (1.004, 1.232)
		≥28	0^b^					0^b^				
	Smoking	Non-smoker	−0.320	0.090	12.624	0.000	0.726 (0.608,0.866)	−0.201	0.083	5.918	0.015	0.818 (0.695,0.962)
		Current	−0.156	0.091	2.911	0.088	0.856 (0.716, 1.023)	−0.190	0.083	5.245	0.022	0.827 (0.703,0.973)
		Ex-smoker	0^b^					0^b^				
	Alcohol	Never	−0.221	0.122	3.268	0.071	0.802 (0.631, 1.019)	−0.197	0.123	2.548	0.110	0.821 (0.645, 1.046)
		Current	−0.289	0.123	5.536	0.019	0.749 (0.588,0.953)	−0.187	0.123	2.308	0.129	0.829 (0.652, 1.056)
		Abstinent from alcohol	0^b^					0^b^			.	
	Physical activity	Unqualified	0.275	0.039	48.487	0.000	1.316 (1.218, 1.422)	0.247	0.043	33.182	0.000	1.280 (1.177, 1.392)
		Qualified	0^b^					0^b^				
	Sedentary behavior	Yes	0.081	0.034	5.538	0.019	1.084 (1.014, 1.160)	0.082	0.037	4.813	0.028	1.086 (1.009, 1.168)
		None	0^b^					0^b^				
	Dietary diversity	Insufficient	0.019	0.061	0.097	0.756	1.019 (0.904, 1.149)	0.097	0.068	2.040	0.153	1.101 (0.965, 1.257)
		Moderate	0.003	0.037	0.008	0.928	1.003 (0.933, 1.080)	0.066	0.043	2.416	0.120	1.069 (0.983, 1.162)
		Sufficient	0^b^					0^b^			.	
Carotid stenosis
	Intercept		0.961	1.548	0.386	0.535		6.604	0.784	70.911	0.000	
	Waistline		0.007	0.011	0.431	0.511	1.007 (0.986, 1.029)	−0.088	0.007	183.349	0.000	0.916 (0.904,0.927)
	Hip circumference		−0.071	0.012	34.274	0.000	0.932 (0.910,0.954)	0.002	0.002	1.757	0.185	1.002 (0.999, 1.006)
	Systolic pressure		−0.001	0.006	0.008	0.927	0.999 (0.988, 1.011)	−0.008	0.004	3.956	0.047	0.992 (0.985, 1.000)
	Diastolic pressure		−0.005	0.008	0.395	0.530	0.995 (0.979, 1.011).	−0.007	0.005	2.002	0.157	0.993 (0.982, 1.003)
	FBG		−0.133	0.070	3.631	0.057	0.875 (0.763, 1.004)	−0.004	0.009	0.208	0.648	0.996 (0.979, 1.013)
	Total cholesterol		0.009	0.006	2.369	0.124	1.009 (0.997, 1.021)	0.000	0.005	0.000	0.989	1.000 (0.989, 1.011)
	TG		0.003	0.008	0.093	0.760	1.003 (0.986, 1.019)	−0.019	0.006	11.076	0.001	0.981 (0.970,0.992)
	HDL-C		0.004	0.011	0.106	0.745	1.004 (0.982, 1.025)	−0.014	0.007	3.707	0.054	0.987 (0.973, 1.000)
	LDL-C		−0.022	0.008	7.613	0.006	0.979 (0.964,0.994)	−0.007	0.004	2.600	0.107	0.993 (0.985, 1.001)
	Gender	Men	−0.085	0.129	0.434	0.510	0.919 (0.714, 1.182)	0.354	0.090	15.387	0.000	1.424 (1.194, 1.700)
		Female	0^b^					0^b^				
	Age	≤ 30	0.045	0.279	0.027	0.871	1.047 (0.606, 1.808)	0.575	0.206	7.791	0.005	1.778 (1.187, 2.663)
		31–49	−0.075	0.255	0.086	0.769	0.928 (0.563, 1.529)	−0.077	0.194	0.159	0.690	0.926 (0.633, 1.353)
		50–64	0.171	0.258	0.438	0.508	1.186 (0.715, 1.967)	0.292	0.196	2.208	0.137	1.339 (0.911, 1.967)
		≥65	0^b^					0^b^				
	BMI	<18.5	0.490	0.489	1.001	0.317	1.632 (0.625, 4.259)	−0.708	0.300	5.569	0.018	0.492 (0.273,0.887)
		18.5–23.9	0.552	0.408	1.831	0.176	1.737 (0.781, 3.864)	−0.537	0.148	13.225	0.000	0.585 (0.438,0.781)
		24–27.9	0.708	0.382	3.430	0.064	2.030 (0.960, 4.292)	−0.584	0.112	27.114	0.000	0.558 (0.448,0.695)
		≥28	0^b^					0^b^				
	Smoking	Non-smoker	0.987	0.588	2.821	0.093	2.683 (0.848, 8.487)	0.077	0.186	0.173	0.677	1.080 (0.750, 1.556).
		Current	1.260	0.588	4.587	0.032	3.525 (1.113, 11.169)	−0.154	0.188	0.671	0.413	0.857 (0.593, 1.239)
		Ex-smoker	0^b^					0^b^				
	Alcohol	Never	0.197	0.590	0.112	0.738	1.218 (0.383, 3.867)	0.161	0.282	0.325	0.569	1.175 (0.675, 2.043)
		Current	0.362	0.591	0.376	0.540	1.437 (0.451, 4.575)	−0.027	0.283	0.009	0.924	0.973 (0.559, 1.695)
		Abstinent from alcohol	0^b^					0^b^				
	Physical activity	Unqualified	0.338	0.132	6.572	0.010	1.402 (1.083, 1.815)	0.076	0.080	0.897	0.344	1.079 (0.922, 1.262)
		Qualified	0^b^					0^b^			.	
	Sedentary behavior	Yes	0.030	0.112	0.072	0.789	1.030 (0.828, 1.282)	0.084	0.073	1.317	0.251	1.087 (0.942,1.255)
		None	0^b^					0^b^				
	Dietary diversity	Insufficient	0.216	0.189	1.309	0.253	1.242 (0.857, 1.799)	0.244	0.146	2.796	0.095	1.277 (0.959, 1.700)
		Moderate	−0.144	0.129	1.249	0.264	0.866 (0.672, 1.115)	0.055	0.082	0.442	0.506	1.056 (0.899, 1.241)
		Sufficient	0^b^					0^b^				

b is the symbol of the statistical result derived from the system.

#### Influencing factors of carotid atherosclerosis without FLD

Compared with participants of age ≥65 years, participants whose age ≤ 30 years (OR: 0.076, 95% CI: 0.063, 0.092, *p* < 0.000) and 31-49 years (OR: 0.218, 95% CI: 0.194, 0.244, *p* < 0.000) were considered protective factors. Compared with participants of BMI ≥ 28, participants with BMI <18.5 was a protective factor (OR: 0.594, 95% CI: 0.448, 0.788, *p* < 0.000). Non-smoking was a protective factor compared with smoking cessation (OR: 0.726, 95% CI: 0.608, 0.866, *p* < 0.000). Current drinkers were less likely to develop carotid atherosclerosis than former drinkers who abstained (OR: 0.749, 95% CI: 0.588, 0.953, *p* = 0.019). Hip circumference was a protective factor, and greater hip circumference was associated with the likelihood of developing carotid atherosclerosis (OR: 0.970, 95% CI: 0.961, 0.979, *p* < 0.000). Both systolic blood pressure (OR: 1.022, 95% CI: 1.019, 1.025, *p* < 0.000) and diastolic blood pressure (OR: 1.005, 95% CI: 1.001, 1.010, *p* = 0.024) were risk factors. Elevated fasting blood glucose was a risk factor (OR: 1.012, 95% CI: 1.005, 1.019, *p* = 0.001). Being male was a greater risk factor than being female (OR: 1.104, 95% CI: 1.024, 1.189, *p* = 0.009); unqualified exercise was a risk factor compared with qualified exercise (OR: 1.316, 95% CI: 1.218, 1.422, *p* < 0.000); and non-sedentary behavior was a risk factor compared with sedentary behavior (OR: 1.084, 95% CI: 1.014, 1.160, *p* = 0.019).

#### Influencing factors of carotid artery stenosis without FLD

Smoking was a risk factor compared with smoking cessation (OR: 3.525, 95% CI: 1.113, 11.169, *p* = 0.032); unqualified exercise was a risk factor compared with qualified exercise (OR: 1.402, 95% CI: 1.083, 1.815, *p* = 0.010). Hip circumference was a protective factor (OR: 0.932, 95% CI: 0.910, 0.954, *p* = 0.000), and LDL-C was a protective factor (OR: 0.979, 95% CI: 0.964, 0.994, *p* = 0.006).

### Influencing factors of carotid disease in participants with FLD

The results of multiple logistic regression analysis in participants with FLD are given in Table 3.

#### Factors influencing carotid atherosclerosis with FLD

Participants of age ≤ 30 years (OR: 0.087, 95% CI: 0.068, 0.112, *p* < 0.000) and 31-49 years (OR: 0.257, 95% CI: 0.223, 0.296, *p* < 0.000) were protective factors compared with participants of age ≥65 years. Non-smoking was a protective factor compared with smoking cessation (OR: 0.818, 95% CI: 0.695, 0.962, *p* = 0.015), and smoking was also a protective factor (OR: 0.827, 95% CI: 0.703, 0.973, *p* = 0.022). Hip circumference was a protective factor (OR: 0.967, 95% CI: 0.958, 0.977, *p* < 0.000); WC was a risk factor (OR: 1.030, 95% CI: 1.022, 1.038, *p* < 0.000); elevated systolic blood pressure was a risk factor (OR: 1.013, 95% CI: 1.010, 1.017, *p* < 0.000); elevated blood glucose was a risk factor (OR: 1.010, 95% CI: 1.004, 1.015, *p* = 0.001); compared with participants with BMI≥28, people with BMI 18.5–23.9 (OR: 1.163, 95% CI: 1.002, 1.351, *p* = 0.047) and BMI 24–27.9 (OR: 1.112, 95% CI: 1.004, 1.232, *p* = 0.041) were more likely to develop carotid atherosclerosis; unqualified exercise was a risk factor (OR: 1.280, 95% CI: 1.177, 1.392, *p* < 0.000); and non-sedentary behavior was also a risk factor (OR: 1.086, 95%CI: 1.009, 1.168, *p* = 0.028).

#### Factors influencing carotid artery stenosis with FLD

Waist circumference was a protective factor (OR: 0.916, 95% CI: 0.904, 0.927, *p* < 0.000); systolic blood pressure was also a protective factor (OR: 0.992, 95% CI: 0.985, 1.000, *p* = 0.047); and elevated TG was a protective factor (OR: 0.981, 95% CI: 0.970, 0.992, *p* = 0.001). Compared to participants with a BMI ≥ 28, participants with BMI <18.5 (OR: 0.492, 95% CI: 0.273, 0.887, *p* = 0.018), 18.5–23.9 (OR: 0.585, 95% CI: 0.438, 0.781, *p* < 0.000), and 24–27.9 (OR: 0.558, 95% CI: 0.448, 0.695, *p* < 0.000) were risk factors. Men were more at risk than women (OR: 1.424, 95% CI: 1.194, 1.700, *p* < 0.000), and age ≤ 30 years was a risk factor (OR: 1.778, 95% CI: 1.187, 2.663, *p* = 0.005).

## Discussion

To investigate the influencing factors of carotid atherosclerosis and stenosis in participants with and without FLD, we investigated the presence of carotid atherosclerosis and stenosis identified in subjects in a large sample of people at health management centers in China. Relevant lifestyle and metabolic factors were also explored. In this study of 47,063 participants, there were 2,8,860 (61.3%) without FLD and 18,203 (38.7%) with FLD. Among the participants without FLD, 23.9% had carotid atherosclerosis and 1.4% had carotid artery stenosis. In participants with FLD, 39.7% had carotid atherosclerosis and 5.8% had carotid artery stenosis.

### Influencing factors of carotid atherosclerosis in participants without FLD

We found that age ≤ 30 years and 31–49 years were protective factors for carotid atherosclerosis compared with age ≥65 years. With increasing age, the prevalence of carotid atherosclerosis increases gradually (15), which may be related to the aging of blood vessels in elderly individuals and the reduction in vascular elasticity in vascular calcification (16). Although the results were statistically significant, the OR value of the factor was very small, which may lead to weak convincing results. In our study, participants with a BMI ≤ 18.5 were less likely to develop carotid atherosclerosis than those with a BMI of ≥28. A cohort study (15) found that long-term high BMI was associated with a carotid atherosclerosis index and plaque volume. The participants without FLD did not necessarily have a lower BMI, so the possibility that a high BMI may increase the risk of carotid atherosclerosis should be considered. Non-smoking was a protective factor for carotid atherosclerosis compared with previous smoking. Studies have shown that active and passive smoking may lead to an increased carotid artery calcification index in patients with essential hypertension (17), and exposure to cigarette smoke appears to be a contributing factor to atherosclerosis. We also found that current drinkers were less likely to develop carotid atherosclerosis than previous drinkers. Previous studies have shown (18) that moderate alcohol consumption was inversely associated with carotid atherosclerosis among Han, Uighur, and Kazakh populations in China. Moreover, compared with the non-drinking elderly, drinking one to six cups per week was negatively correlated with carotid atherosclerosis (19). Therefore, most of the non-fatty liver patients in our study may be moderate drinkers. Compared with women without FLD, men were more likely to develop carotid atherosclerosis. Studies have shown (20) that reduced social support and lack of awareness of the disease and physiological differences between the sexes contribute to differences in the prevalence of carotid atherosclerosis. Therefore, we should not only be aware of the differences between men and women in carotid artery disease but also provide different treatment measures. We also found that, compared with qualified exercise, unqualified exercise was a risk factor for carotid atherosclerosis; however, non-sedentary behavior was a risk factor for carotid atherosclerosis compared with sedentary behavior. Physical activity levels were significantly and negatively correlated with cIMT (21). The risk of the carotid artery and carotid plaque (CP) abnormalities decreased significantly with increased exercise levels, and the negative correlation was stronger among participants aged ≥60 years. However, sedentary leisure time was not associated with cIMT or CP. Physical activity is important for carotid artery health, especially in the elderly. Research has shown that self-reporting can underestimate the actual amount of time taken by some sedentary behaviors and thus cannot be considered the gold standard, while a combination of self-reporting and usage of devices that objectively assess sedentary behavior may be more accurate (22, 23).

The results showed that the larger the hip circumference, the less likely the carotid atherosclerosis development. Hassinen et al. (24) found that the smaller the hip circumference, the faster the progression of carotid atherosclerosis. We found that higher systolic and diastolic blood pressure were associated with a greater risk of carotid atherosclerosis in participants without FLD. Studies suggested that the brachial muscle and systolic blood pressure index were associated with increased cIMT (25). The target organ damage and incidence of cardiovascular and cerebrovascular events significantly increase in hypertensive patients with abnormal blood pressure rhythm (26), which increased the risk of carotid atherosclerosis. Therefore, blood pressure should be controlled at not only a normal level but also at the morning peak of blood pressure. Elevated fasting glucose was a risk factor for carotid atherosclerosis, which was consistent with the results of previous studies (27). Although participants did not have FLD, elevated fasting glucose may represent endocrine disorders, resulting in the decreased metabolic function of individuals and an increased possibility of atherosclerosis.

### Influencing factors of carotid artery stenosis in participants without FLD

Our results found that smoking and unqualified exercise were risk factors for carotid artery stenosis, which was identical to carotid atherosclerosis, suggesting that smoking and lack of exercise may be risk factors for carotid disease. Hip circumference was a protective factor for carotid stenosis in patients without FLD. Earlier studies (28, 29) have shown that hip circumference was negatively associated with type 2 diabetes and CVD morbidity and mortality. We found that low-density lipoprotein was a protective factor for carotid atherosclerosis in patients without FLD. However, studies (30) showed that increased LDL-C levels were an independent risk factor for carotid artery stenosis. The difference may be due to the difference in subjects.

### Factors influencing carotid atherosclerosis in patients with FLD

Patients with age ≤ 30 and 31–49 years were protective factors for carotid atherosclerosis compared with patients with age ≥65 years. Young and middle-aged people were less likely to develop carotid atherosclerosis, which was consistent with participants without FLD. The possible reason may be that aging is a process characterized by progressive loss of tissue and organ functions (31), ROS-induced damage causes age-related functional loss, and this oxidative stress is also involved in age-related diseases. Compared with those who had quit smoking, non-smoking was a protective factor, which was consistent with those without FLD. However, smoking was also a protective factor. The possible reason may be that smoking was a risk factor for carotid artery abnormalities, but there exists a dose-dependent relationship (32, 33). Therefore, it is necessary to further explore the specific amount of smoking, such as the number of carotid artery influences, to better guide smokers to gradually change their smoking habits. We also found that those with BMI of 18.5–23.9 and 24–27.9 were more likely to develop carotid atherosclerosis. In a cohort study of NAFLD patients in the United States (34), more than 10% of participants were thin, and Asians made up almost half of the thin people with FLD. The possible reason may be that Asians with fatty liver may be more emaciated due to physical differences, so participants with FLD with a lower BMI may be more prone to carotid atherosclerosis. We also found that unqualified exercise and sedentary time of up to 5 h were risk factors. A large study of Lavie et al. (35) sedentary times revealed that 49,493 adults living in 20 countries sat for an average of 5 h a day, and studies of older adults found that 59% sat for 4 h a day, and 27% sat for 6 h a day (13). In this study, according to self-reports, sedentary behavior time ≤ 5 h was a risk factor for carotid atherosclerosis in participants with FLD. Self-reported assessments of sitting time may vary across fields, backgrounds, and countries.

In participants with FLD, the greater the hip circumference, the less likely carotid atherosclerosis development, which was consistent with the participants without FLD, suggesting that hip circumference may be a protective factor for carotid atherosclerosis, regardless of whether participants had FLD. WC was a risk factor for carotid atherosclerosis, which was not found in people without FLD. Studies have shown that in diabetic patients (36), a larger WC increases the burden of carotid atherosclerosis. It may also be that WC was generally larger in people with FLD than in people without FLD. In a Chinese cohort study (37), increased WC and sustained high WC were found to be associated with increased cIMT. Therefore, maintaining normal WC may be important to promote vascular health. Studies (38) have shown that curcumin supplementation may have a positive effect on visceral fat and abdominal obesity associated with FLD. Therefore, curcumin supplementation may be considered for people with large abdominal fatty liver. Elevated systolic blood pressure and elevated blood sugar levels were risk factors for carotid atherosclerosis, similar to participants without FLD. A 5-year follow-up of a Korean male occupational population showed that the incidence of hypertension in moderate and severe fatty liver patients was 1.60 times and 2.22 times higher than that in the control group (8). After adjusting for age, BMI, liver function, blood lipids, smoking and other factors, FLD was still correlated with hypertension.

### Influencing factors of carotid stenosis in patients with FLD

People with BMI <18.5, 18.5–23.9, and 24–27.9 were less likely to have carotid artery stenosis, which was consistent with previous studies (39, 40). Men were more likely to develop carotid artery stenosis than women, which was consistent with the influencing factors of carotid atherosclerosis. In patients with fatty liver, those aged ≤ 30 years were more likely to develop carotid artery stenosis than those aged ≥65 years, and increasing age was an independent risk factor for carotid artery stenosis (30). Studies have shown that (4) FLD may be a more important contributor to subclinical atherosclerosis in younger, rather than older, populations. In our study, the possible reason may be that among the participants with FLD, the elderly died due to carotid artery stenosis. This may also be due to the small proportion of the two age-groups.

We found that the larger the WC, the less likely the carotid stenosis development. There was a statistically significant difference in the prevalence of high cIMT between WC 79cm and WC < 79cm (41), and the optimal WC cutoff currently used to diagnose carotid artery disease may be lower than Japan's current diagnostic criteria. Other studies (42) have shown that WC in Shanghai women was significantly correlated with cIMT, and WC ≥85cm can be used as a risk indicator for subclinical carotid artery disease. Therefore, more evidence should be compiled to determine the most reliable thresholds for carotid atherosclerosis risk. The higher the systolic blood pressure, the less likely the presence of carotid stenosis, which was not found in participants without FLD. The prevalence of baseline characteristics and vascular risk factors in our study population differs from previous studies (43). Elevated TG was a protective factor for carotid artery stenosis. The relationship between TG and CVD risk factors has been controversial. Hypertriglyceridemia was often associated with lipoprotein changes, such as decreased HDL and HDL-C levels and increased non-HDL-C levels, all of which were associated with increased cardiovascular risk (44). Therefore, more studies are needed to explore the mechanism between elevated TG levels and carotid artery disease.

There were several limitations in our study. First, lifestyle characteristics were collected through questionnaires. Although self-report can help judge the background status of an individual at that time, the form of self-report may lead to information bias. Therefore, a combination of objective instrument-based measurements and self-reporting may lead to more accurate results. Second, the influencing factors of carotid atherosclerosis and stenosis in patients with and without fatty liver were only discussed through cross-sectional investigation, but the comparison between the two groups and the discussion on the longitudinal influence of carotid artery disease were lacking, which should be remedied in future to better prevent and control the occurrence and development of carotid artery disease. Third, this study only discussed the influencing factors of carotid artery disease in participants with and without FLD but did not discuss the type and severity of fatty liver; therefore, the type and severity of fatty liver should be further clarified to further explore the risk factors for carotid artery disease.

## Conclusion

The prevalence of FLD was 38.7% in the health check-up population in China. In participants without FLD, 6,885 (23.9%) suffered from carotid atherosclerosis and 407 (1.4%) suffered from carotid artery stenosis. In participants with FLD, 7,227 participants (39.7%) had carotid atherosclerosis and 1,055 participants (5.8%) had carotid stenosis. The lifestyle and metabolic factors of carotid atherosclerosis and carotid stenosis were different in the patients without and with FLD and mainly focused on exercise, sedentary behavior, smoking, alcohol consumption, hip circumference, and blood pressure. Our study investigated lifestyle and metabolic factors in a large sample of participants without and with FLD, which can provide a basis for the targeted prevention of carotid disease risk and lay a foundation for the study of CVD risk factors.

## Data availability statement

The original contributions presented in the study are included in the article/supplementary material, further inquiries can be directed to the corresponding author/s.

## Ethics statement

The studies involving human participants were reviewed and approved by research and development of the health coaching technology intervention decision support system on residents healthy lifestyle self-reporting (No: 2020-S587). Written informed consent was obtained from all participants for their participation in this study.

## Author contributions

CZ: conceptualization. CZ, JW, SD, GG, LL, YL, AC, and ZC: funding acquisition. CZ, JW, SD, GG, LL, YL, ZC, YD, JX, and AC: writing—review and editing and investigation. JW and YD: formal analysis. YD and JX: writing—original draft. All authors contributed to the article and approved the submitted version.

## Funding

This work was supported by the Special Funding for the Construction of Innovative Provinces in Hunan (No. 2020SK53618).

## Conflict of interest

The authors declare that the research was conducted in the absence of any commercial or financial relationships that could be construed as a potential conflict of interest.

## Publisher's note

All claims expressed in this article are solely those of the authors and do not necessarily represent those of their affiliated organizations, or those of the publisher, the editors and the reviewers. Any product that may be evaluated in this article, or claim that may be made by its manufacturer, is not guaranteed or endorsed by the publisher.
